# The interplay between Chinese EFL teachers’ positive psychological capital and their work engagement

**DOI:** 10.1016/j.heliyon.2023.e13151

**Published:** 2023-01-21

**Authors:** Jieping Xu

**Affiliations:** School of Foreign Languages, Chaohu University, Hefei, China

**Keywords:** Positive psychological capital, Work engagement, Language learning, EFL Context

## Abstract

Positive psychology has recently gained a great momentum, which per se motivated us to scrutinize the correlation between teachers’ positive psychological capital (TPPC) and their work engagement (WE). The correlation between these two variables has been discovered to be significant as it can create a friendly educational ambiance for the learners to passionately learn. Although several studies have been conducted regarding the aforementioned variables separately or with regard to some other variables, a study on their bond and importance has remained unaddressed. Therefore, the aim of this study is to find the relationship between TPPC and their WE. The participants of this study were 412 Chinese teachers (including 82 males, and 330 females) of different ages and levels of education. Two online instruments were utilized in this study, namely Positive Psychological Capital Scale and the Utrecht Work Engagement Scale (UWES). The results showed a positive and significant association between these two variables. The implications of this study are finally discussed in light of previous studies. Some implications are also offered to EFL educators.

## 1 Introduction

Teaching has always been considered a stressful, demanding job because many psychological problems are associated with it [1]. Positive psychology started as a new type of psychology in 1998 when it was chosen by Martin Seligman as the theme for his term as president of the American Psychological Association. It is a reaction against past practices, which focused on mental illness, maladaptive behavior, and negative thinking. Stress can be perceived as detrimental when it comes to both the teaching and learning process and to reduce stress, psychological capital should be raised in teachers. Hence, when stress is felt by teachers while working and when they feel ill-being, it will negatively affect students’ academic and behavioral outcomes [2,3]. Recent attention has centered on positive psychological capital in teachers [4], and a growing number of these studies suggested that teacher frustration could affect work engagement (WE) and lead to mental health problems [5,6,7]. The two variables in this study can be delineated as follows: according to Luthans et al. [8], Positive psychological capital consists of four factors: Hope, optimism, effectiveness, enjoyment, grit, and resilience that enable teachers to improve their own well-being [9,10]. WE is also defined as work-related states of mind that include energy, dedication, and preoccupation [11].

Consequently, further research in the area of positive psychological capital is needed, even though the radical role of the teacher has gained attention and is seen as a key component of second language acquisition [12]. It also plays a central role in teacher well-being for both teacher engagement and student academic success [13]. This study is of great benefit since it shifts the attention from students toward teachers' well-being. This is the rationale behind conducting the present study. More specifically, first, as far as the researcher knows, there are no studies on the interaction between TWE (Teacher's Engagement in Work) and TPPC (Teacher's Positive Psychological Capital). Second, no such study using the variables mentioned has been conducted in China. Third, examining the interaction between these two variables helps authorities to strengthen the education system. Finally, a few studies have been conducted on teachers in this area that is the reason why teachers' WE and PPC are of great significance.

Moreover, considering previous research, some strengths and weaknesses can be identified. As for the cons, no studies have been conducted to find interactions between these two variables investigated in this study. So PPC can be analyzed with another variable or alone. It is crucial because the more engaged and dedicated teachers are, the more they can encourage students to pursue their academic goals, thus facilitating the learning process and thereby strengthening the educational system. Second, WE has been previously studied with several negative factors such as anxiety and burnout, which have been suggested. When instructors feel on edge and burned out, they are less likely to be effectively involved in what they do and they cannot be imaginative to come up with unused thoughts for their educating strategies and empowering their understudies. Hence, it can adversely impact the instructive framework in which instructors play an urgent part. There has been a move from negative feelings to positive ones after consideration has been driven to studies about PPC. In this regard, this research might be of extraordinary significance since the relationship between these two factors have been considered. But any previous research that paved the way for new research with new results can still be useful. Therefore, this study aims to answer the following question.

1. Is there any significant association between Chinese EFL teachers' positive psychological capital and teacher's work engagement?

## 2 Background

### 2.1 EFL teachers’ positive psychological capital

Positive mental capital came into the presence when the positive organizational behavior hypothesis has come into consideration. The organizational environment causes numerous issues. Agreeing with Luthans, Avolio, Avey, and Norman [8], PPC comprises four factors: hope, adequacy, resilience, and positive thinking, which makes it conceivable for instructors to increase their well-being. Hope hypothesis was concocted by Snyder's [14], which places emphasis on effective agency and pathways that can be perceived as significant with respect to the hope hypothesis, meaning that they are enthusiastic to realize their objectives which have been arranged sometime recently. It has been found that exceedingly confident people implemented the errands superior in games, mental alterations, psychotherapy, and in some other situations [15,16,18].

At that point, the hope hypothesis was connected within the instructive space due to its unimaginable noteworthiness [19]. As a result, it has been proposed that cheerful instructors are profoundly likely to see students' misbehaviors as the openings through which unused techniques for overseeing the lesson can be attempted. In other words, instructors with high levels of trust have the capability to handle the obstacles, which piece coming with their objectives as restricted to their partners with moo trust [20].

Another subfactor of PPC that can be taken into account is self-efficacy, which is characterized when one believes in his abilities to obtain particular accomplishments [21]. Numerous components have been affected by this component, for illustration, the way they make effort to reach their objectives, the strongness, and determination they have when facing an issue, the tolerance they have when confronting an extreme circumstance like a disappointment, and how stressed they are as they are pressurized [22]. This component has drawn consideration since it can massively influence schools and students' lifestyles. Considering it, self-efficacy in teachers is characterized as the degree to which a teacher believes that he/she can affect his/her students' performance. Subsequently, the attempt the instructors have when educating, the way the objectives are set and the reasonability of them, the excitement and passion that they put into practice, are all impacted by teachers' adequacy. Instructors with a better sense of adequacy are perceived to have more energy and more determination toward their work and it is highly likely for them to remain as instructors for their whole lives [21]. Having this in mind, self-efficacy can be regarded as an important factor for instructors because in this way many positive behaviors in educating can emerge, leading to affluent results for learners [17] [22] [18,23,24]. Instructors having more viability are believed to endure more as experiencing issues and managing less persuaded students. According to Gibson and Dembo (1984), this viable instruction affects such students’ development. In addition, new experiences are more welcomed and appear more committed [25,26].

Another factor of PPC that can be taken into thought is being resilient, which is conceptualized as one’ capacity to deal with misfortunes and adapt with the stress to make a more grounded and more joyful individual after a troublesome circumstance [8]. Educator versatility is one of the components that results in solidness in several angles of a teacher's work [27,28,29,30]. The resilience of teachers is the capability to adjust to various circumstances as well as boost their competence when facing awful conditions [31]. Based on what Kitching, Morgan, and O'Leary [32] postulate, a few factors empower instructors to develop rather than fair survival in their occupations. In addition, instructors, who are resilient, are found to form more commitment to their organizations and they are more likely to assist their colleagues when they are faced with a problem, construct an inviting working climate, tolerate inconveniencies without making complaints, and fulfill with their employments [33]. It has moreover been pointed out that objectives can be better accomplished, superior choices can be made, and a cheerful, profitable, and solid life can be procured by resilient instructors [34].

Finally, positive thinking is the last factor of PPC. Positive thinking was coined by Scheier and Carver [35] that is perceived as a positive approach to life [36] and it is additionally accepted that positive thinking or optimism is attributed to the way individuals address trouble. What is of great importance in some research studies is that idealistic instructors are exceptionally likely to be more viable in their educating since they can take more risks. They, in expansion, create a positive viewpoint of their students and see impediments as novel challenges that can be overcame [37].

Psychological capital has been found to have a positive correlation with the degree to which people are committed to their organizations, and the amount to which people are satisfied with their jobs [38,39]. It recognizes an individual's strengths and the areas in which he/she is superior and healthy. People find psychological capital of great benefit because it causes them to be more productive and stronger [40]. Furthermore, people can be helped by PPC, which fosters acclimatization to their environment, mitigate their stress, tackle their difficulties, and lead a fulfilled life. Therefore, it can be viewed as a contributory factor to determine how well a piece of work is performed and also as a predictor for workers' attitudes towards their work [41].

Another study conducted by Khajavy et al. [42] indicated that language classrooms are impacted by psychological capital, which comprises four constructs, self-efficacy, hope, optimism, and resilience. The role of psychological capital in learners’ second/foreign (L2) willingness to communicate (WTC), L2 motivational self-system, and L2 achievement was studied. The findings showed that psychological capital positively predicted those three constructs. Hence, its role can be significantly important in education. Another study conducted by Viseu et al. [43] revealed that teacher motivation can be improved, and they can feel satisfied with their job when they grow their psychological capital. The significance of teacher satisfaction on their motivation was underlined and it emphasized the relationship between teacher motivation and PPC. Moreover, another study carried out by Luthans et al. [8] showed that there was a significantly positive association considering four facets of psychological capital and satisfaction and performance, meaning that teachers, who have a great psychological capital, are more satisfied with their jobs and perform better.

### 2.2 Teachers’ work engagement

According to Schaufeli et al. [11], WE is defined as a mental state, which is relevant to work and comprises energy, devotion, and immersion [11,29]. To put it simply, how energetic and agile a teacher is and how dedicated he is toward his job leads to immersion, which means to what extent he is immersed in his job and can do his job with alacrity. Psychologists' attention has been attracted by WE as there is a growing interest in positive psychology [30,[44], [45], [46]]. It should be taken into account that WE was previously studied with negative emotions such as burnout, anxiety, etc. Despite the fact that these days it is tremendously preferred to deal with WE along with positive aspects pertinent to success and fulfillment at work [9,47]. In this respect, burnout is viewed as the opposite to WE because teachers make an endeavor to dedicate their energy and time to implementing tasks, while burnout is defined as a state that teachers are no longer interested in their jobs, and cannot feel devoted to what they do. Internal and external factors can affect teachers' work [48]. According to Han and Wang [5], WE consists of three components: vigor, dedication, and absorption. It should be noted that there is a widening difference between WE and workaholism in that the former one is a positive attribute, contributing to positive results and the latter causes more damage, leading to burnout. As has been mentioned, vigor was one of the components of WE through which it can be perceived that teachers' quality and WE are influenced by teachers’ intrapersonal variables such as self-efficacy [5].

In a recently published study, the key role of well-being, comprising the domains of positive emotion, engagement, relationships, meaning, and achievement, in mediating the relationship between Psychological Capital and the classroom management of teachers of English as a foreign language (EFL) was explored. It was fund that the well-being of EFL teachers plays a significant role in mediating the relationship between their psychological capital and classroom management though [49]. Another study showed that job commitment and academic optimism are good predictors of EFL Chinese teachers’ psychological capital [50].

## 3 Method

All procedures performed in the study were in accordance with the ethical standards of the Chaohu University research committee and with the 1964 Helsinki declaration and its later amendments or comparable ethical standards. Informed consent was obtained from all the individual participants included in this study.

### 3.1 Participants

Data collection commenced on November 1st, 2021 and lasted 11 days. The participants were 412 randomly chosen teachers (82 males and 330 females) whose age groups varied from 22 to 60. They mainly came from Anhui (N = 251) and other cities. Regarding their academic degrees, it varied from bachelor's degree to doctor's degree (bachelor's: 129; master's 233; doctor's 40). Among these EFL teachers, 31 worked in primary school, 97 work in high school, 229 in college or university, 44 in vocational school, and 11 in other institutes (Table 1).

**Table 1:** Participants demographics.

Background Information	No.
**Gender** Male	82
Female	330
**Academic degrees**	
Bachelor	129
Master	233
Doctor	50
**Schools**	
Primary school	31
High school	97
Vocational school Other institutes College & university	44 11 229

### 3.2 Instruments

The Positive Psychological Capital Scale developed by Tösten and Özgan [51] was used to determine how positive psychological capital is perceived by teachers. The scale comprises the following sub-factors: Self-efficacy (4 items), Optimism (5 items), Trust (4 items), Extroversion (5 items), Psychological Resilience (5 items), and Hope (3 items). The scale was prepared using a 5-point Likert type response method from never [1] to totally participating [5]. Reliability analysis was done by Tösten and Özgan [51]. Cronbach's Alpha values were calculated as 0.80 for self-sufficiency, 0.80 for optimism, 0.83 for confidence, 0.79 for extroversion, 0.76 for psychological durability, 0.73 for hope, and each sub-dimension as 0.93 [52].

In order to measure WE, the short form of the Utrecht Work Engagement Scale (UWES) was utilized [53]. The UWES features three dimensions of engagement: Vigor (3 items), Dedication (3 items), and Absorption (3 items). It is a five-point scale in which descriptive statements were needed.

### 3.3 Procedures

In order to answer the research question, the valid questionnaires were distributed online through Wenjuanxing (an online platform used to make questionnaires) via Wechat, QQ or network linking, and data were collected smoothly. In order for the trustworthiness of this study to be guaranteed, the respondents knew about their rights to withdraw from the research for whatever reason they have. When they started, they were apprised of making validated responses and felt assured that their private information was only utilized to answer the research question. Altogether, 411 valid questionnaires were gleaned from different primary schools, high schools, vocational schools, language institutes, colleges, and universities in China. As the participants made no contact with the researcher, there were no conflicts of interest between the researcher and the respondents. Then, the gathered responses were checked again for possible errors before being processed by SPSS software for further statistical analysis. In the final step, the questions of this research were responded according to the collected data.

### 3.4 Data analysis

In the present study, to find the relationship between TPPC and teachers' WE, Spearman Rho Correlation was utilized which showed that TPPC is positively and significantly correlated with teachers’ WE.

## 4 Results

### 4.1 Primary data analysis

In order to feel assured about the instruments reliability, unwavering quality of the rebellious utilized for information collection, the analyst ran a Cronbach Alpha test for each survey. Table 2 appears that all three surveys, TPPC and TWE had palatable Cronbach Alpha lists (0.951, and 0.958, individually).

**Table 2:** Reliability of the questionnaires.

Questionnaires	Cronbach's Alpha	N of Items
Teacher's Positive Psychological Capital	.951	26
Teacher's Work Engagement	.958	16

In order to check if the data are needed to be analyzed parametrically or not, the researcher ran a test of normality. Table 3 shows the results of K–S, which reveals that the data are normal (0.200) for TPPC, but the data are not normal (0.005) for TWE. Thus, the non-parametric analysis was run for measuring the relationship between the variables by applying a Spearman Rho correlation index.

**Table 3:** Test of normality.

	Kolmogorov-Smirnov			Shapiro-Wilk		
	Statistic	df	Sig.	Statistic	df	Sig.
TPPC	.027	412	.200	.991	412	.010
TWE	.055	412	.005	.980	412	.000

The participants were tabulated based on their gender, regarding their scores on TWE and TPPC. The mean scores of male and female participants were very close to each other regarding their TWE scores (61.62, 60.38), while this difference increased in TPPC scores (103.95, 99.51). Table 4 shows that the groups are almost homogeneous regarding their standard deviation (11.93, 11.45) for TWE and (15.13, 15.26) for TPPC.

**Table 4:** Descriptive statistics of male and female participants.

	gender	N	Mean	Std. Deviation	Std. Error Mean
TWE	male	82	61.62	11.93	1.31
	female	330	60.38	11.45	.63
TPPC	male	82	103.95	15.13	1.67
	female	330	99.51	15.26	.84

The research question is concerned with the presence of any association between Chinese EFL teachers' positive psychological capital and teacher's work engagement. To this conclusion, after making beyond any doubt that the information are not typical, a Spearman Rho test was run.

Spearman Rho was used to find the association between the factors. Table 5 illustrates that teachers' positive psychological capital and teachers' work engagement are directly correlated (0.782), which suggests that the higher list of teachers' positive psychological capital, the higher the amount of teachers' work engagement. Besides, the importance level for this relationship is .000, which suggests that there's a direct and noteworthy relationship between the TPPC and TWE.

**Table 5:** Correlations between teachers' positive psychological capital and Teacher's work engagement.

			TWE
Spearman's rho	TPPC	Correlation Coefficient	.782**
		Sig. (2-tailed)	.000
		N	412

## 5 Discussion

This study aimed to scrutinize the relationship between TPPC and their WE. The results showed that there is a direct and significant relationship between the TPPC and TWE. It has been believed that students can be highly affected by their teachers both mentally and educationally since they play a paramount role in every aspect of students' life. Hopes would be dashed to learn a new language if the students were not treated fairly and if the teachers did not have the capability to convey the subject-matter in an appropriate way. It has been reported that some students have lost their motivation due highly to the fact that a new language could not have been taught in an easy, understandable way. As it has been said, a good teacher is the one, who can teach materials in the easiest way and it could not be achieved unless the teacher himself comprehends the materials thoroughly. It is not, therefore, beneficial for teachers if they tend to keep their profiles to make the materials complicated because one of the rare factors, which urge students to stay motivated is the amount to which they think they are able to understand the materials, otherwise they will feel abased, their stress cannot be abated, and they find it abstruse to achieve a new language. The root cause of students abhorring a new language lies in the way they have been taught. As a result, teachers’ well-being is a contributory factor to enable students to feel extrinsically motivated and confident to acquire a new language. On the other hand, teachers themselves feel highly engaged in their work and they are incredibly motivated to challenge themselves and their students in an effort to find better results in the process of teaching. As a matter of fact, it should be accentuated that PPC makes it accessible for teachers to win acclaim and gain the respect of their students. In terms of the research method, to strengthen both the reliability and validity of this study, this research method has been applied in the current study.

The result, which has been achieved, is in line with the following research studies. PPC and its components are positively correlated with TWE. It has recently been preferred to make an association between WE and positive emotions which fosters job satisfaction [47,48]. On the other hand, the results are in congruence with these studies [20,24,30,54] because TPPC highly affects the way teachers treat their learners, and the way teachers see the obstacles and problems. This finding indirectly corroborates the ideas of Pena, Rey, and Extremera [55], who proposed that negative feelings could be better managed by teachers, who have more capability to withstand terrible conditions in their classes. As mentioned by Han and Wang [5], vigor, as one of the components of work engagement, affects teachers' quality, meaning that the more vigorous the teachers are, the more engaged they are in what they do and as a result of which both teachers and students can feel better. This study posits that TPPC and WE can both improve teachers’ quality and resilience, which is in line with the study conducted by Luthans et al. [8] that highlighted resilient teachers, who strived hard to overcome their obstacles and also their stress are highly likely to be happier, influencing their work. Because resilience is a component of positive psychological capital. The findings reflect those of Khajavi et al. [42], who mentioned language classrooms are influenced by psychological capital including self-efficacy, hope, optimism, and resilience. The justification is that all these four facets urge the teachers to do their best and encourage the students to be actively engaged in what they do. The point which was revealed by Viseu et al. [43] was that teacher motivation can be enhanced and they can feel satisfied with their job when they grow psychological capital, meaning that job satisfaction and WE are highly interwoven that they cannot be separated and an increase in one of them leads to a rise in another one. Hence, as mentioned, both of these are facilitated by psychological capital; accordingly, it is the reason why the referred study has something in common with the present study. The results of the current study lend support to that of study conducted by Yildiz [38] in that it has been proven that psychological capital plays a very important role in the degree to which people are committed to their organizations, and the amount to which people are satisfied with their jobs. Therefore, it can be inferred that WE shows how much a person is committed to his job and satisfied with his job. In other words, WE stems from job satisfaction and commitment.

Based on the results of this research, PPC stresses six metrics: Self-Efficacy, Positive Thinking, Certainty, Extraversion, Mental Resistance, and hope. Two factors of this study were positively correlated with each other. Self-efficacy is reflected in several aspects of a teacher's life. They have confidence in whatever they do which is relevant to their job. This gives them confidence in what they are doing and gives strong persuasion to their students, as students outwardly feel persuaded to learn something new.

Another component that might be considered is that they know what they need to do to be successful, suggesting that they not only know their objectives but also are aware of achieving those aims. This gives the trainers full conviction of themselves, which drives them to engage effectively in what they are doing. Good faith, one of the metrics of PPC, can be outlined using a few examples. Positive instructors feel vibrant. Being idealistic enables trainers to live their working life positively and it contributes to seeing disappointment as a risk factor, so that they are never locked in facing issues, making them more grounded.

Certainty is a measurement of TPPC, as well. Certain instructors are aware of their competent duties. This implies that their commitments can be fulfilled by trainers and what makes them feel fulfilled in their work is that they can actually fulfill their commitments in case there are some issues. Surely the instructors are too enthusiastic to resolve the learners’ problems. The reticent instructors cannot deal with their feelings and their pessimistic thoughts cannot be controlled, so they are constantly pushed. Consequently, this does not let learners tackle their problems. In the same way, if the students do not address the issues of appreciating the learning process, they feel demotivated, and the teachers themselves feel unfulfilled in their work. Instructors, who are confident, believe that they are competent enough in their work, so it is highly likely for them to feel sure in whatever they do.

Extraversion that is a factor of TPPC can be shown as follows: If they face an issue in their work, they are courageous and confident to consult the experts if it is necessary to resolve the problems. Extroverted people have a tendency to talk about their problems so as to find a solution.

Extroversion can also be taken into account. Teachers, who are extroverted can have creative modern thoughts for their work and their penchant for straightforwardness in their proficient lives. Open-minded people do not feel stubborn and one-sided, and they have the heart to tune into the thoughts that do not agree with the ones they have. Consequently, they can come up with original thoughts after giving others kinds of thoughts enough contemplation. There is no doubt that teaching may be a job that requires ingenuity, and without which teachers might not have been so wonderfully fruitful. The more imaginative a trainer is, the more committed they are to their work. Extroverted instructors tend to show honesty and directness with their learners, because trustworthiness is the most important requirement for teaching.

Furthermore, resilience can be emphasized. When teachers overcome pessimistic thoughts purely for educational purposes and devote their energy to educating students, they are more likely to withstand defeat. They also think of solutions to unexpected problems, simply because they are able to face difficulties. They do not see these problems as obstacles, but they learn from them and that keeps them interested in their work and their things. They have more commitment in their lives. Furthermore, difficult situations are regarded as challenges, which enables them to welcome solving the problems. It would be very important for a sufficiently motivated teacher not to be easily irritated when faced with problems. The commitment to work in this way depends largely on the teachers’ mindset and how they view their problems. Hope, as another component of TPPC, can be considered as the core of psychological resistance. Aspiring instructors describe their difficulties as an experience that makes them mature and gives them help to rebuild their personality. Some problems have to be dealt with at the same time complicating the situation and making teachers ready to face their own challenges. Work and work actively. All of the above dimensions show that there is a positive correlation between TPPC and TWE and therefore the results of this study are really significant.

## 6 Conclusion

This study was carried out in order to analyze the association between TPPC and WE among 412 Chinese teachers. It was shown that there is a direct significant correlation between TPPC and TWE in that TPPC has an important impact on the amount of engagement that is made by them. This study can be beneficial for both teachers and students in that teachers should be mentally healthy to be satisfied with what they do and to be actively engaged and engrossed in their work since their work-life is as significant as their personal life and both of which are interrelated. Hence, an increase in one of them leads to a rise in another.

Secondly, students can find this research beneficial, as well because whenever teachers' issues are taken into consideration, the first group that can be affected are learners, who can find motivation or demotivation in their class. Therefore, making a friendly and supportive atmosphere in which students feel relaxed and motivated and teachers are well-adjusted seems necessary. Last but not least, the authorities, who are responsible for strengthening education infrastructures can be influenced by this study, too in that the four attributes of PPC; *hope*, *resilience*, *self-efficacy*, and *optimism* can be developed in teachers by them, using different ways such as, giving teachers promotion or raise, providing them with regular sessions in which they can boost their well-being, insuring them so as to increase their job satisfaction, and paying attention to their problems and allocating some budgets to them in order to improve teachers’ mental health.

Like other studies, this research is limited in some ways. So, some new studies for avid researchers are left to be conducted. First of all, given the lack of time and words, this study could not test the relationship between each of the sub-factors TPPC, each of them seems important to be studied with WE. PPC is a new topic to which attention has recently been paid and all its dimensions solely can help teachers to be stronger in what they do as their job. Secondly, even though this study was quantitative and teachers’ minds cannot be probed through numbers, qualitative research should be conducted in which open-ended questions can be posed and diaries can be kept to better understand how teachers think. It is also of great significance to conduct longitudinal studies. Last not least, the three dimensions of WE (i.e., vigor, absorption, and dedication) can be further explored in L2 contexts focusing on other psychological factors related to teachers.

## Author contribution statement

Jieping Xu, doctor: Conceived and designed the experiments; Performed the experiments; Analyzed and interpreted the data; Contributed reagents, materials, analysis tools or data; Wrote the paper.

## Funding statement

This work was sponsored by the key University-level pedagogical project funded by Chaohu University (Grant No. ch21jxyj08), China.

## Data availability statement

Data will be made available on request.

## Declaration of interest’s statement

The authors declare no competing interests.
